# Public support for standardised packaging and policy implementation: an analysis of European survey data

**DOI:** 10.1093/eurpub/ckag001

**Published:** 2026-01-28

**Authors:** Rui Liang, Anthony A Laverty, Ariadna Feliu, Cristina Martinez, Armando Peruga, Constantine Vardavas, Filippos T Filippidis

**Affiliations:** Public Health Policy Evaluation Unit, School of Public Health, Imperial College London, London, United Kingdom; Public Health Policy Evaluation Unit, School of Public Health, Imperial College London, London, United Kingdom; Public Health Policy Evaluation Unit, School of Public Health, Imperial College London, London, United Kingdom; Center for Biomedical Research in Respiratory Diseases (CIBER en Enfermedades Respiratorias, CIBERES), Madrid, Spain; Center for Biomedical Research in Respiratory Diseases (CIBER en Enfermedades Respiratorias, CIBERES), Madrid, Spain; Tobacco Control Unit, Cancer Control and Prevention Program, WHO Collaborating Center On Tobacco Control, Institut Català d’Oncologia, l’Hospitalet de Llobregat, Barcelona, Spain; Cancer Control and Prevention Group, Institut d’Investigació Biomèdica de Bellvitge-IDIBELL, l’Hospitalet de Llobregat, Barcelona, Spain; Department of Public Health, Mental Health, and Maternal and Child Health Nursing, School of Nursing—Bellvitge Campus, Universitat de Barcelona, Barcelona, Spain; Philip R. Lee Institute for Health Policy Studies, University of California San Francisco, San Francisco, CA, United States; Center for Biomedical Research in Respiratory Diseases (CIBER en Enfermedades Respiratorias, CIBERES), Madrid, Spain; Tobacco Control Unit, Cancer Control and Prevention Program, WHO Collaborating Center On Tobacco Control, Institut Català d’Oncologia, l’Hospitalet de Llobregat, Barcelona, Spain; Cancer Control and Prevention Group, Institut d’Investigació Biomèdica de Bellvitge-IDIBELL, l’Hospitalet de Llobregat, Barcelona, Spain; Laboratory of Epidemiology, Hygiene and Medical Statistics, School of Medicine, National and Kapodistrian University of Athens, Athens, Greece; Public Health Policy Evaluation Unit, School of Public Health, Imperial College London, London, United Kingdom

## Abstract

We conducted a secondary analysis of Eurobarometer survey data from 27 European countries, collected in 2017 (*n* = 28 300) and 2023 (*n* = 26 358), to assess changes in public support for standardised tobacco packaging. In the pooled analysis, support remained unchanged in the 19 MS without relevant legislation (adjusted Prevalence Ratio [aPR]=0.94, 95% Confidence Interval: 0.86–1.03), whereas the 8 MS that had implemented the policy by 2023 were significantly more likely to experience an increase in support (interaction term: aPR = 1.28, 1.17–1.41). The increase in support offers reassurance to policymakers advocating for tobacco packaging regulations and encourages MS to consider the adoption of similar policies.

## Introduction

Standardised (sometimes known as ‘plain’) packaging of tobacco is a recent policy development to stem the harms of tobacco. Since implementation in Australia in 2012, more than 20 countries have adopted this measure. Standardised packaging aims to reduce smoking prevalence and, hence, tobacco-related health problems by reducing the visual appeal of tobacco brands and strengthening health warnings [1].

Public support is always important to enact policy change, and this is particularly true considering tobacco industry opposition to standardised packaging [2]. Research suggests significant increases in public support for standardised packaging policies where these have been implemented, although existing studies have mostly focused on individual countries or specific points in time [3], meaning there is a need for longitudinal analyses and comprehensive transnational evaluations. This study examines changes in public support for standardised packaging across 27 European Union (EU) Member States (MS) between 2017 and 2023.

## Methods

We conducted a secondary analysis of data from 27 EU MS, collected during two Eurobarometer survey waves: wave 87.1 (March 2017, *n* = 27 901) and wave 99.3 (May–June 2023, *n* = 26 358). Eurobarometer collects data through multi-stage sampling, and data are weighted so that they are representative of the population aged ≥15 years at country and EU level [4]. Data collection for both 2017 and 2023 was conducted through face-to-face interviews in the local language.

The outcome variable was support for tobacco standardised packaging. Participants were asked: *‘Would you be in favour or against any of the following measures? Introducing “plain packaging” for cigarettes, that is, standardised packaging with a fixed colour and design and the removal of all branding (such as images and corporate logos)?’*. Responses were: ‘In favour’, ‘Against’, and ‘Don’t know’. We classified the responses into two categories: ‘In favour’ and ‘Not in favour’ ('Against’ or 'Don’t know’).

Demographic characteristics collected included age (15–24, 25–39, 40–54, ≥55 years), gender (male, female), area of residence (rural, urban), difficulty paying bills in the last 12 months (almost never/never, from time to time, most of the time), age at completion of full-time education (0–15, 16–19, 20+ years, still studying), and smoking status (never smoker, tried/used in the past, current smoker).

Country-level policy status was classified based on the timing of legislation and full implementation, creating three groups: implemented, legislated but not yet implemented, and no legislation. In 2017, France had already implemented the policy, Ireland had legislated but not yet implemented the policy, while all other included EU MS had no legislation. By 2023, Ireland, Slovenia, Belgium, the Netherlands, Hungary, Denmark, and Finland had implemented standardised packaging policies.

We used a multilevel Poisson regression model to account for the clustering of responses within countries. The exposure variable was the policy status of tobacco standardised packaging in each MS, analysed as an interaction term with the year of the survey. The model was adjusted for all covariates listed above. As no EU MS has implemented standardised packaging for e-cigarettes, the analysis did not account for e-cigarette use and focused solely on tobacco. We also ran individual Poisson regression models, adjusted for the sociodemographic variables mentioned above and for smoking status, in each country individually. We present results as adjusted prevalence ratios (aPRs) with 95% confidence intervals (CIs).

## Results

**Figure 1.**
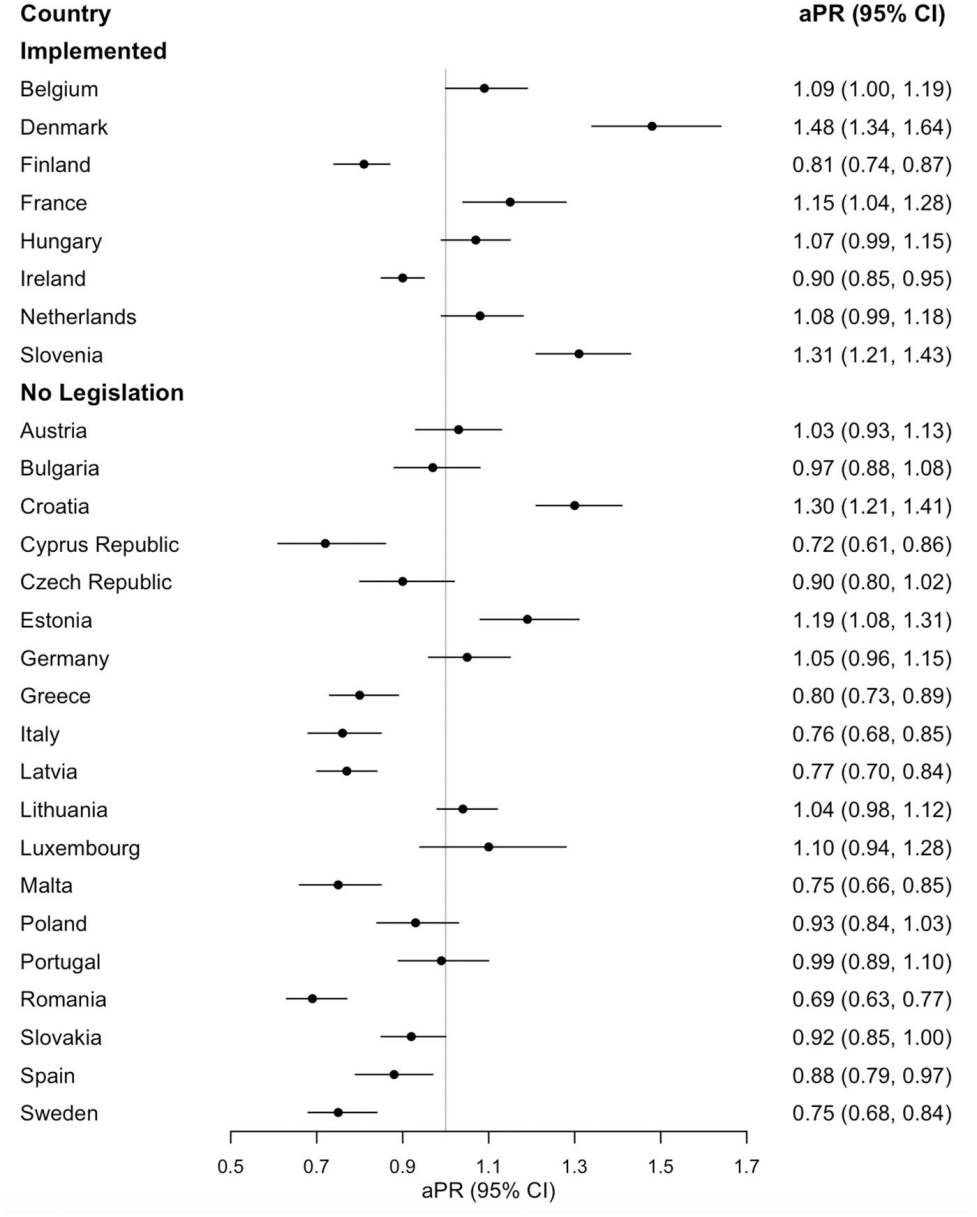
Change in public support for the tobacco standardised packaging policy by European Union Member State from 2017 to 2023.

Sociodemographic characteristics of the sample are shown in Table S1. Across the EU, 43.5% of respondents (95% CI: 42.5–44.5) were in favour of standardised packaging in 2017 and 42.4% (41.5–43.4) in 2023 (Table S2). Figure 1 illustrates the change in support for tobacco standardised policies between 2017 and 2023 in each of the 27 EU MS. Six MS (four of which had implemented the policy) showed statistically significant increases in support, with aPRs ranging from 1.09 in Belgium to 1.48 in Denmark. In contrast, ten MS experienced statistically significant declines in support (two of which had implemented the policy), with aPRs ranging from 0.69 in Romania to 0.90 in Ireland. The remaining two of the countries that implemented the policy (Hungary and the Netherlands) showed borderline non-significant increases in support (Table S3).

In pooled analyses support for standardised packaging was significantly more likely to have increased in MS that implemented the policy by 2023 compared to the MS without relevant legislation (aPR = 1.28, 1.17–1.41) (Table S4). There were no changes in support among countries not implementing the policy (aPR = 0.94, 0.86—1.03).

Overall, support for standardised packaging policies was significantly higher among individuals who completed their education at age 20 or older (aPR = 1.06, 1.01—1.11) and those who were still studying (aPR = 1.06, 1.01—1.13), compared to those who completed their education at age 15 or younger. In contrast, support was significantly lower among former (aPR = 0.93, 0.89–0.96) and current smokers (aPR = 0.74, 0.69–0.79) when compared to never smokers.

## Discussion

This study found that public support for standardised packaging policies increased on average across the eight countries that implemented this measure. However, there was some variation by country: four MS that implemented the policy saw statistically significant increases, two others showed borderline non-significant increases, whereas two experienced decreases in public support. This average increase across the implementing MS aligns with previous studies and provides additional support for the positive impact of policy implementation on public support for standardised packaging [5–7]. Policy advocacy and public education are effective ways to communicate the public health benefits of standardised packaging [8]; countries may experience increased activity in these areas before and immediately after the introduction of the policy. Increased public support could also be attributed to the positive effects of policy implementation, including reduced product attractiveness and perhaps lower smoking prevalence [9]. Nevertheless, the support change was not uniform across all MS, indicating that the implementation of the policy itself cannot fully explain the phenomenon; local factors may also alter the views of the population regarding the policy.

The study also showed that individuals with higher educational levels were more likely to favour standardised packaging, which is consistent with many health behaviours. In contrast, support was lower among both former and current smokers. Warning labels and standardised packaging may reduce the perceived enjoyment of smoking and generate resistance to this policy [10]. As such, targeted advocacy and health education strategies based on the characteristics of different populations may be utilised to improve understanding and support for policy among key groups.

We used Eurobarometer data, which covers 27 EU MS, is nationally representative and designed to enable cross-national comparisons. However, further work could use more than the two data points employed here, including data collected over shorter intervals to better determine the dynamics of policy support.

We combined both country-level and individual-level factors (e.g. education, gender, and age) using multilevel Poisson regression analyses. Further research could employ qualitative insights into the underlying reasons for public support or lack thereof for standardised packaging. Additionally, e-cigarettes and heated tobacco products were not included in the analysis, although they may influence public views on policy, as could the broader tobacco control environment of each country.

## Conclusion

This study identifies a significant increase in public support in countries that have already implemented standardised packaging. While the myriad factors which drive public support underscore the need for multisectoral approaches, these results provide confidence for policymakers considering similar policies in their settings.

Key points

- Support for standardised packaging of tobacco products across the European Union was 43.5% in 2017 and 42.4% in 2023.
- Support for standardised packaging was significantly more likely to have increased in Member States that implemented the policy by 2023 compared to those without relevant legislation.
- Among the eight Member States that implemented the policy, four experienced a significant increase in support, two a borderline non-significant increase and two a decrease.
- Support for standardised packaging was significantly lower among former and current smokers.

## Authors contribution

FTF and AAL conceived the study. RL had the main role in data analysis and preparation of the initial draft. All authors contributed to data interpretation, reviewed and edited the manuscript. FTF is the guarantor of the data used in this analysis.

## Supplementary Material

ckag001_Supplementary_Data

## Data Availability

The Eurobarometer datasets are owned by the European Commission and are partially or wholly publicly available at www.gesis.org.
