# Supplementary material for: Public support for standardised packaging and policy implementation: an analysis of European survey data
Source: Eur J Public Health. 2026 Jan 28;36(2):ckag001. doi: 10.1093/eurpub/ckag001 (PMC13064488; doi:10.1093/eurpub/ckag001)
Supplement: ckag001_Supplementary_Data [file ckag001_supplementary_data.docx]

**Table S1: Included sample across 27 European Union Member States in 2017 and 2023**

| **Category** | **2017 N (weighted %, CI)** | **2023 N (weighted %, CI)** | **Total N (weighted %, CI)** |
| --- | --- | --- | --- |
| **Gender** |  |  |  |
| Woman | 14659 (51.9, 50.9 - 52.9) | 14122 (51.5, 50.5 - 52.5) | 28781 (51.7, 51.0 - 52.4) |
| Man | 11896 (48.1, 47.1 - 49.1) | 12179 (48.5, 47.5 - 49.5) | 24075 (48.3, 47.6 - 49.0) |
| **Age** |  |  |  |
| 15-24 years | 2346 (12.6, 11.9 - 13.4) | 2386 (12.3, 11.6 - 13.1) | 4732 (12.5, 12.0 - 13.1) |
| 25-39 years | 5385 (22.9, 22.1 - 23.8) | 5170 (22.0, 21.2 - 22.9) | 10555 (22.5, 21.9 - 23.1) |
| 40-54 years | 6424 (25.1, 24.2 - 26.0) | 6577 (25.1, 24.2 - 25.9) | 13001 (25.1, 24.5 - 25.7) |
| 55 years and older | 12400 (39.4, 38.4 - 40.3) | 12223 (40.6, 39.6 - 41.5) | 24623 (40.0, 39.3 - 40.7) |
| **Area of Residence** |  |  |  |
| Rural | 8662 (30.7, 29.8 - 31.7) | 8972 (31.4, 30.5 - 32.3) | 17634 (31.1, 30.4 - 31.7) |
| Urban | 17880 (69.3, 68.3 - 70.2) | 17377 (68.6, 67.7 - 69.5) | 35257 (69.0, 68.3 - 69.6) |
| **Difficulties in Paying Bills** |  |  |  |
| Almost never/never | 16773 (64.3, 63.3 - 65.3) | 17328 (65.8, 64.8 - 66.7) | 34101 (65.1, 64.4 – 65.8) |
| From time to time | 6742 (26.7, 25.8 - 27.6) | 6803 (26.5, 25.6 - 27.4) | 13545 (26.6, 26.0 – 27.2) |
| Most of the time | 2599 (9.0, 8.5 - 9.6) | 2043 (7.7, 7.2 - 8.3) | 4642 (8.4, 8.0 - 8.7) |
| **Age at Completion of Full-time Education** |  |  |  |
| 0-15 years | 3993 (17.4, 16.6 - 18.2) | 2968 (13.5, 12.9 - 14.3) | 6961 (15.4, 14.9 - 15.9) |
| 16-19 years | 11291 (41.4, 40.4 - 42.4) | 11188 (43.5, 42.5 - 44.5) | 22479 (42.5, 41.8 - 43.2) |
| 20+ years | 9185 (31.5, 30.6 - 32.5) | 9858 (33.3, 32.3 - 34.2) | 19043 (32.4, 31.8 - 33.1) |
| Still Studying | 1696 (9.7, 9.0 - 10.4) | 1972 (9.7, 9.1 - 10.4) | 3668 (9.7, 9.2 - 10.2) |
| **Smoking Status** |  |  |  |
| Never | 14000 (52.6, 51.5 - 53.6) | 14322 (55.7, 54.7 - 56.7) | 28322 (54.2, 53.5 - 54.9) |
| Tried/Used in the past | 5833 (20.0, 19.2 - 20.8) | 5683 (19.6, 18.9 - 20.4) | 11516 (19.8, 19.3 - 20.4) |
| Current | 6670 (27.5, 26.5 - 28.4) | 6317 (24.6, 23.8 - 25.5) | 12987 (26.0, 25.4 - 26.6) |

**Table S2: Weighted change (in percentage) in public support for standardised packaging policy between 2017 and 2023.**

|  | **Weighted % in favour (95% CI)** | |
| --- | --- | --- |
|  | **2017** | **2023** |
| No legislation | 44.0 (43.0 to 45.0) | 39.2 (38.1 to 40.4) |
| Policy legislated* | 73.2 (70.2 to 75.9) | - |
| Policy implemented | 39.0 (35.6 to 42.4) | 51.1 (49.1 to 53.1) |
| All 27 countries | 43.5 (42.5 to 44.5) | 42.4 (41.5 to 43.4) |

*There were no countries which legislated standardised packaging between 2017 and 2023 and had not implemented the policy at the time of the 2023 survey.

**Table S3:** **Change in public support for tobacco standardised packaging policy by European Union Member State from 2017 to 2023.**

| **Country** | **adjusted Prevalence Ratio** | **95% Confidence Interval** |
| --- | --- | --- |
| **Implemented** |  |  |
| Belgium | 1.09 | 1.00 - 1.19 |
| Denmark | 1.48 | 1.34 - 1.64 |
| Finland | 0.81 | 0.74 - 0.87 |
| France | 1.15 | 1.04 - 1.28 |
| Hungary | 1.07 | 0.99 - 1.15 |
| Ireland | 0.90 | 0.85 - 0.95 |
| Netherlands | 1.08 | 0.99 - 1.18 |
| Slovenia | 1.31 | 1.21 - 1.43 |
| **No Legislation** |  |  |
| Austria | 1.03 | 0.93 - 1.13 |
| Bulgaria | 0.97 | 0.88 - 1.08 |
| Croatia | 1.30 | 1.21 - 1.41 |
| Cyprus Republic | 0.72 | 0.61 - 0.86 |
| Czech Republic | 0.90 | 0.80 - 1.02 |
| Estonia | 1.19 | 1.08 - 1.31 |
| Germany | 1.05 | 0.96 - 1.15 |
| Greece | 0.80 | 0.73 - 0.89 |
| Italy | 0.76 | 0.68 - 0.85 |
| Latvia | 0.77 | 0.70 - 0.84 |
| Lithuania | 1.04 | 0.98 - 1.12 |
| Luxembourg | 1.10 | 0.94 - 1.28 |
| Malta | 0.75 | 0.66 - 0.85 |
| Poland | 0.93 | 0.84 - 1.03 |
| Portugal | 0.99 | 0.89 - 1.10 |
| Romania | 0.69 | 0.63 - 0.77 |
| Slovakia | 0.92 | 0.85 - 1.00 |
| Spain | 0.88 | 0.79 - 0.97 |
| Sweden | 0.75 | 0.68 - 0.84 |

**Table S4: Poisson regression model across 27 European Union Member States exploring the association between standardised packaging policy implementation and changes in support for the policy (2017-2023).**

| **Variable** | **aPR** | **95% CI** | **p-value** |
| --- | --- | --- | --- |
| **Year** | | | |
| 2017 | Ref. | Ref. | Ref. |
| 2023 | 0.94 | 0.86 - 1.03 | 0.162 |
| **Policy Status** | | | |
| No legislation | Ref. | Ref. | Ref. |
| Legislated but not implemented | 1.26 | 1.11 - 1.43 | <0.001 |
| Implemented | 0.93 | 0.82 - 1.05 | 0.215 |
| **Year # Policy Status** | | | |
| 2017 # No legislation | Ref. | Ref. | Ref. |
| 2023 # Legislated but not implemented* | - | - | - |
| 2023 # Implemented | 1.28 | 1.17 - 1.41 | <0.001 |
| **Area of Residence** | | | |
| Rural | Ref. | Ref. | Ref. |
| Urban | 1.01 | 0.97 - 1.06 | 0.521 |
| **Difficulties in Paying Bills** | | | |
| Almost never/never | Ref. | Ref. | Ref. |
| From time to time | 0.98 | 0.93 - 1.02 | 0.310 |
| Most of the time | 1.02 | 0.94 - 1.11 | 0.566 |
| **Age at Completion of Full-time Education** | | | |
| 0-15 years | Ref. | Ref. | Ref. |
| 16-19 years | 1.04 | 0.99 - 1.08 | 0.102 |
| 20+ years | 1.06 | 1.01 - 1.11 | 0.029 |
| Still Studying | 1.06 | 1.01 - 1.13 | 0.028 |
| **Gender** | | | |
| Female | Ref. | Ref. | Ref. |
| Male | 0.99 | 0.96 - 1.02 | 0.632 |
| **Age** | | | |
| 15-24 years | Ref. | Ref. | Ref. |
| 25-39 years | 1.08 | 1.04 - 1.13 | <0.001 |
| 40-54 years | 1.03 | 0.98 - 1.09 | 0.224 |
| 55 years and older | 1.01 | 0.96 - 1.07 | 0.644 |
| **Smoking Status** | | | |
| Never | Ref. | Ref. | Ref. |
| Tried/Used in the Past | 0.93 | 0.89 - 0.96 | <0.001 |
| Current | 0.74 | 0.69 - 0.79 | <0.001 |

*There were no countries which legislated standardised packaging between 2017 and 2023 and had not implemented the policy at the time of the 2023 survey.
